# Investigating the Performance of Asphalt Modified with Rubber Powder and Surface Organic Layered Double Hydroxides

**DOI:** 10.3390/gels12070641

**Published:** 2026-07-17

**Authors:** Chenze Fang, Xu Guo, Yuanzhao Chen, Zhenxia Li, Tengteng Guo, Hui Li, Jingyu Yang, Haijun Chen, Qi Chen, Chaohui Wang, Qian Chen, Xiaoyan Han, Yi Lu

**Affiliations:** 1 School of Civil Engineering and Transportation, North China University of Water Resources and Electric Power, Zhengzhou 450045, China; 2 Technology Innovation Center of Henan Transport Industry of Utilization of Solid Waste Resources in Trafffc Engineering, North China University of Water Resources and Electric Power, Zhengzhou 450045, China; 3 Henan Province Engineering Technology Research Center of Environment Friendly and High-Performance Pavement Materials, Zhengzhou 450045, China; 4 Department of Transportation, Southeast University, Nanjing 210096, China; 5 School of Highway, Chang’an University, Xi’an 710064, China; 6 Faculty of Infrastructure Engineering, Dalian University of Technology, Dalian 116024, China; 7 School of Foreign Languages, North China University of Water Resources and Electric Power, Zhengzhou 450045, China

**Keywords:** road engineering, rubber powder, surface organicized LDHs, rheological properties, micromechanisms

## Abstract

In order to promote the sustainable development of road engineering, this study used waste tire rubber powder (RP) and surface organic layered double hydroxide (SOM-LDHs) to modify 70# matrix asphalt. The Box–Behnken design response surface method with three factors (rubber powder content, surface organic layered double hydroxide content, shear temperature) and three responses (penetration, ductility, softening point) was used to optimize the preparation parameters. The optimum formula was determined to be 21.7% rubber powder content, 4.8% surface organic layered double hydroxide content, and 160 °C shear temperature. The effect of the modifier on the surface morphology was analyzed using a rotating film oven test and ultraviolet aging test. The high and low temperature rheological properties of asphalt were evaluated by dynamic shear rheometer (DSR), bending beam rheometer (BBR), and the multi-stress creep recovery test (MSCR). The microstructure was observed by scanning electron microscopy (SEM) and atomic force microscopy (AFM). The aging mechanism was investigated by Fourier transform infrared spectroscopy (FTIR) and gel permeation chromatography (GPC). The results show that after aging, the complex shear modulus of rubber powder/surface organic layered double hydroxide composite modified asphalt is the highest, which is 27.35% higher than that of matrix asphalt. The rutting factor reaches 79.86 kPa at 46 °C, the phase angle decreases by 11.83% after UV aging, and the high temperature plastic deformation resistance is the best. In the low temperature range of −18 °C to −24 °C, the creep stiffness of the composite modified asphalt is about 30% lower than that of the matrix asphalt, while the m value is increased by about 15%, and the low temperature stress relaxation performance is significantly improved. The strain recovery rate of composite modified asphalt under 3.2 kPa stress reaches 78.5%, and the unrecoverable creep compliance is as low as 0.18 kPa^−1^, which is better than that of matrix asphalt and single rubber powder modified asphalt.

## 1. Introduction

Traditional asphalt pavement materials in practical application have some disadvantages, since in high temperature they are easy to soften and to deform, resulting in rutting of the road surface. In the long-term ultraviolet irradiation and oxidation, traditional asphalt materials are easy to crack [1]. The growing global concern for environmental protection and sustainable development will promote modified asphalt to obtain a broader application prospect by virtue of its eco-friendly characteristics. Rubber powder, as an effective modifier, can strengthen its ability to resist permanent deformation and enhance the overall toughness of asphalt pavement [2], and the surface of the organicized LDHs has excellent UV-blocking properties, which can effectively shield UV rays, impede the penetration of oxygen, and significantly enhance the aging resistance of asphalt [3]. In view of this, this study will focus on the surface organicization of LDHs to further enhance the stability of LDHs, their physical properties, high-temperature storage stability, resistance to UV aging, and rheological properties of asphalt, and elucidate the interaction mechanism between the two modifiers and the asphalt matrix, which can provide innovative ideas and theoretical support for the development of pavement materials with both excellent road performance and durable weathering resistance. It also elucidates the interaction mechanism between the two modifiers and the asphalt matrix, which can provide innovative ideas and theoretical support for the development of asphalt pavement materials with excellent road performance and long-lasting weathering resistance. This study is of great theoretical value and practical significance to enhance the service quality of asphalt pavements.

In recent years, LDHs have been applied to the field of asphalt materials as a new type of modifier, which opens up a new direction for asphalt modification research. Li et al. [4,5,6] have confirmed that the layered structure of LDHs effectively prevents ultraviolet light from penetrating into the interior of asphalt. Liu et al. [7] have prepared asphalt films containing LDHs and those that do not, and evaluated the absorbance, reflectance, transmittance, and microscopic morphology of LDH thin films with the aid of a UV-Visible spectrophotometer and a fluorescence microscope to assess the absorbance, reflectance, transmittance, and microscopic morphology of asphalt films. When the doping of LDHs was 5 wt%, its modified asphalt was able to effectively absorb and reflect UV rays, which enhanced the UV aging resistance of the asphalt. At present, the methods of organic modification of LDHs can be categorized into organic modification and intercalation modification [8]. Organic modification is the use of hydroxyl reactive groups on the surface of LDHs to react with other functional groups that have large negative electronegativity to obtain surface organicized LDHs [9]. Wang et al. [10] used LDHs with a mass fraction of 3% and LDHs modified by organicization with different magnesium-to-aluminum ratios, and found that LDHs modified by organicization made the asphalt show better anti-aging performance, which can effectively reduce the degree of aging of the asphalt’s self-healing properties. Xu et al. [5] discussed the change effect of rheological properties of modified asphalt after ultraviolet aging. Their results showed that OLDHs-modified asphalt (OLMA) possesses better UV aging resistance and high temperature stability. Compared with ordinary LDHs, OLDHs show a stronger modification effect, which is mainly attributed to the organic intercalation structure that improves the compatibility between LDHs and the asphalt matrix, thus enhancing the modification efficiency.

The practical application of waste tire modified rubber powder asphalt began in the 1980s. With the enhancement of environmental awareness and the development of resource reuse technology, the treatment of waste tires has gradually attracted attention. The core of this study is dedicated to the in-depth exploration of wet rubber powder modified asphalt. Li et al. [11,12] explored the performance and the interaction mechanism of the intrinsic link between the rubber powder and the modified asphalt, finding that the physicochemical properties of the rubber powder, the mixing process parameters, and its pretreatment method significantly affected the performance of the modified asphalt. The interaction between rubber powder and the asphalt matrix mainly involves swelling and thermo-oxidative degradation processes. When reaching the equilibrium state of asphalt expansion, the prepared rubber powder modified asphalt shows the optimal road performance. Ye et al. [13] found that rubber powder/PPA composite modified asphalt was prepared by adding polyphosphoric acid (PPA), rubber powder, and matrix asphalt as raw materials. The results of conventional performance tests showed that the composite addition of PPA and rubber powder could effectively improve the low-temperature performance of asphalt. However, with the increase in rubber powder, the aging resistance of the composite asphalt gradually decreased, and its storage stability also tended to decline. In contrast, the introduction of PPA enhances the aging resistance of asphalt, keeping its storage stability unaffected. Zhou et al. [14] prepared high viscosity composite modified asphalt. The test showed that 5% SBS, 13% rubber powder, and 4% high viscosity agent were the optimal ratio. The composite modified asphalt at this dosage has good high-temperature rutting resistance, temperature-sensitive performance, and storage stability, and there is physical co-mingling and chemical reaction between each component and the matrix asphalt. Xu et al. [15], in the SBS doping of 4.5%, set the rubber powder doping at 0%, 6%, 10%, and 14% to prepare composite modified asphalt. The results show that the rubber powder made the asphalt structure finer and more dispersed, the mechanical properties were enhanced, the elasticity and deformation resistance of the asphalt was improved, and the adhesion and Young’s modulus were improved. Rubber powder has no significant effect on the degradation of the SBS molecular chain. Ling et al. [16] found that the CRMA binder modified by LDHs showed significantly improved performance in resisting UV-induced degradation.

In summary, the addition of rubber powder can give asphalt good flexibility and anti-fatigue properties, while the unique laminated structure and light shielding properties of LDHs can significantly improve the aging resistance and high temperature stability of asphalt due to its unique layer structure and UV shielding properties [17,18,19]. The composite of rubber powder and LDHs for asphalt modification is expected to realize the complementarity of the performance advantages of the two, and provide a new way to develop high-performance composite modified asphalt. At present, the research in this field has achieved some milestones, mainly focusing on the changes in macroscopic performance indexes, including the softening point increment (SPI), residual needle penetration ratio (PRR), ductility retention ratio (DRR), and its high and low temperature rheological behavior. However, the potential interactions between layered dimetallic hydroxides (LDHs), rubber powder modified asphalt (CRMA) and asphalt matrix, and how these interactions lead to improved aging resistance, have not yet been systematically explained in the current research. The literature has amply demonstrated that asphalt inherently possesses a sol-gel colloidal structure [20,21]. As a road engineering material, the primary focus of this study is on the macroscopic performance of asphalt in road applications, rather than on the characterization of the precise sol-gel chemical transformation process. In view of the above, this study focuses on composite modified asphalt by combining RP and SOM-LDHs. The organic modification of LDHs enhances their compatibility with the asphalt matrix, enabling them to act as physical crosslinking nodes within the system, while the swollen rubber powder forms a continuous elastic gel phase. This hierarchical sol-gel network is expected to improve the rheological properties, thermal stability, and UV aging resistance of asphalt. By systematically investigating the formation mechanism, microstructure, and performance evolution of this gel-like system, this study provides new insights into the design of high-performance, durable asphalt materials from the perspective of sol-gel chemistry.

## 2. Results and Discussion

### 2.1. Optimization of Formulation and Basic Performance

Based on the test results corresponding to the test program proposed in DE8, 13 different parameter combinations corresponding to three different parameters were obtained based on the BBD response surface method design. To ensure the reliability and stability of the model, five tests were repeated at the center of the parameter corresponding to the design. According to the “Highway Engineering Asphalt and Asphalt Mixture Testing Procedures” (JTG E20-2011) [22], the test results are shown in Table 1.

**Figure 1:**
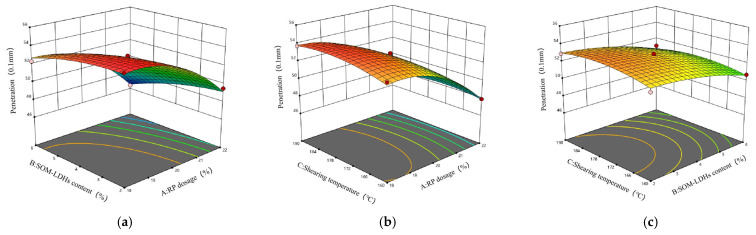
The impact diagram of the interaction of various factors on penetration degree. (**a**) 3D plot of response surfaces of factors A and B interacting with each other; (**b**) 3D plot of response surfaces of factors A and C interacting with each other; (**c**) 3D plot of response surfaces of factors B and C interacting with each other.

**Figure 2:**
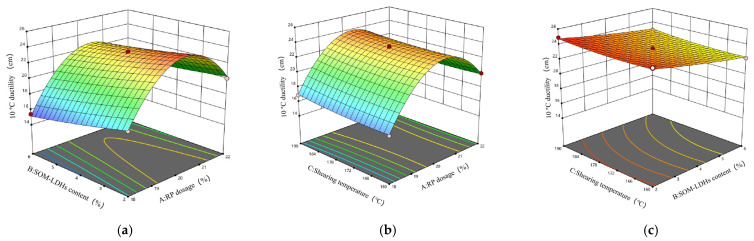
The impact diagram of the interaction of various factors on ductility. (**a**) 3D map and contour plot of response surface for interactions of factors A and B; (**b**) 3D map and contour plot of response surface for interactions of factors A and C; (**c**) 3D map and contour plot of response surface for interactions of factors B and C.

**Figure 3:**
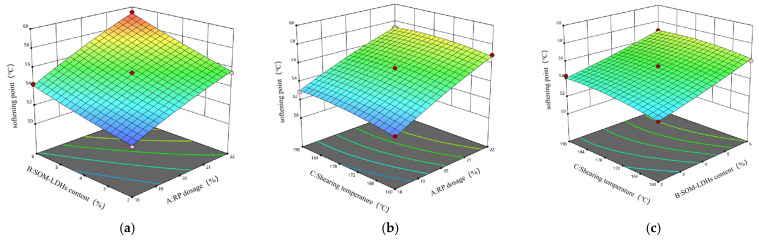
The influence diagram of the interaction of various factors on the softening point. (**a**) 3D map and contour plot of response surface for interactions of factors A and B; (**b**) 3D map and contour plot of response surface for interactions of factors A and C; (**c**) 3D map and contour plot of response surface for interactions of factors B and C.

Based on the 17 sets of experimental data shown in Table 1, the model equations were derived by analyzing the ANOVA for each response output variable, using the response surface design method to assess the significance of the selected model and each variable. Where RP doping is represented by code A, SOM-LDHs doping is represented by code B, and shear temperature is represented by code C. The response surface modeling of RP doping, SOM-LDHs doping, and shear temperature versus needle penetration, ductility, and softening point was obtained by fitting the model equations based on the response surface of needle penetration, as shown in Figure 1, Figure 2 and Figure 3.

Figure 1a shows that the slope of curve A is significantly higher than that of curve B, which indicates that the effect of rubber powder incorporation on penetration is greater than the contribution of SOM-LDHs. Figure 1b further confirms that the larger the rubber powder doping, the smaller the needle penetration. The slope of curve A is higher than that of curve C, which shows that the effect of rubber powder incorporation on asphalt penetration is more significant than that of temperature change. Figure 1c shows that the response surface 3D plot is more gentle than in Figure 1a,b, and the surface does not change much, which indicates that the effect of the interaction of SOM-LDHs doping and shear temperature is not very significant on the experimental results, and the correlation is not strong.

Figure 2a shows that curve A is significantly steeper than curve B, indicating that the effect of RP doping on the ductility is greater than that of SOM-LDHs doping. However, when the RP doping exceeds about 20%, the asphaltene content increases notably, resulting in an increase in molecular chain cohesion, which restricts its extension and slippage in the material tensile test stage, and the plastic deformation ability of the material is weakened. As seen in Figure 2b, curve C is close to a straight line, indicating that the effect of shear temperature on the ductility of modified asphalt is smaller than that of the rubber powder dosage. Combined with Figure 2c, this suggests that, at low temperatures where ductility begins to increase, from the mobility of the asphalt in rubber-modified asphalt increases, molecular movement accelerates during the tensile process, and the rubber powder can be better assist the asphalt molecular chains in stretching and slipping, which is conducive to improving the ductility.

Figure 3a shows that the degree of undulation of curve A is greater than curve B, indicating that the effect of RP doping on the softening point is greater than that of SOM-LDHs doping. Figure 3b shows that the softening point of composite modified asphalt increases significantly with the increase in RP content, while the temperature effect curve (curve C) shows a slight upward trend, indicating that the shear temperature also has a certain effect on the softening point of modified asphalt. Combined with Figure 3c, this shows that with the increase in shear temperature, the softening point of modified asphalt has a tendency to slowly increase, the high temperature of the rubber powder dissolution and crosslinking can be coordinated with the SOM-LDHs chemical adsorption effect, and the rubber powder can be used to improve the softening point. LDHs chemical adsorption can help build a more solid, more restrictive composite structure of asphalt molecules, so that the asphalt can only be softened at higher temperatures, which leads to an increase in the softening point with increasing shear temperature.

The multiple regression models of penetration, ductility, and the softening point were established based on the significant influencing factors, and the equations were shown as (1), (2) and (3), respectively.

(1) $$Needle\;penetration=52.78-2.8A-1.04B+0.1375C-0.275AB-0.325AC-0.1BC-1.69A^{2}-0.465B^{2}+0.615C^{2}$$

(2) $$Ductility=23.3+1.65A-0.95B+0.25C+0.45AB-0.1AC+0.1BC-5.23A^{2}+0.125B^{2}+0.225C^{2}$$

(3) $$Softening\;point=55.34+2.25A+1.5B+0.4C+0.325AB-0.125AC-0.125BC-0.2325A^{2}+0.1175B^{2}-0.3325C^{2}$$

Referring to the technical specifications proposed in the Technical Guide for Design and Construction of Rubber Asphalt and Mixture [23], and based on the simulation and optimization results of the response surface method, the optimal preparation parameters of RP/SOM-LDHs composite modified asphalt are as follows: rubber powder (RP) content 21.7%, SOM intercalated LDHs (SOM-LDHs) content 4.8%, and shear temperature 160 °C. According to this optimization scheme, composite modified asphalt samples were prepared and three major indicators were tested. As shown in Table 2, the measured index values are compared with the optimal values predicted by the model. The results show that the deviations between the two are less than 4%, which verifies that the model has high accuracy in predicting the basic performance changes of asphalt under different modification conditions. Compared with 70# matrix asphalt, the comprehensive performance of RP/SOM-LDHs composite modified asphalt is significantly improved: the ductility value is greatly increased, indicating that the flexibility and deformation resistance of the material are significantly enhanced, penetration is decreased slightly, and the softening point shows only a moderate increase. The above results show that the modification of RP and SOM-LDHs effectively optimizes the road performance of asphalt.

**Table 2:** Performance verification of composite modified asphalt under optimal dosage.

Index	Needle Penetration (0.1 mm)	Softening Point (°C)	10 °C Ductility (cm)
Predicted value	48.0	20.6	57.3
Measured value	47.8	20.3	58.1
70 # base asphalt	65.2	51	30.4

In summary, the optimized preparation parameters of RP/SOM-LDHs composite modified asphalt optimized by the response surface method were 21.7% RP doping, 4.8% SOM-LDHs doping, and 160 °C shear temperature.

### 2.2. Macroscopic Performance Analysis of Aging and Rheological Behavior

#### 2.2.1. Rotating Film Oven Test Results

**Figure 4:**
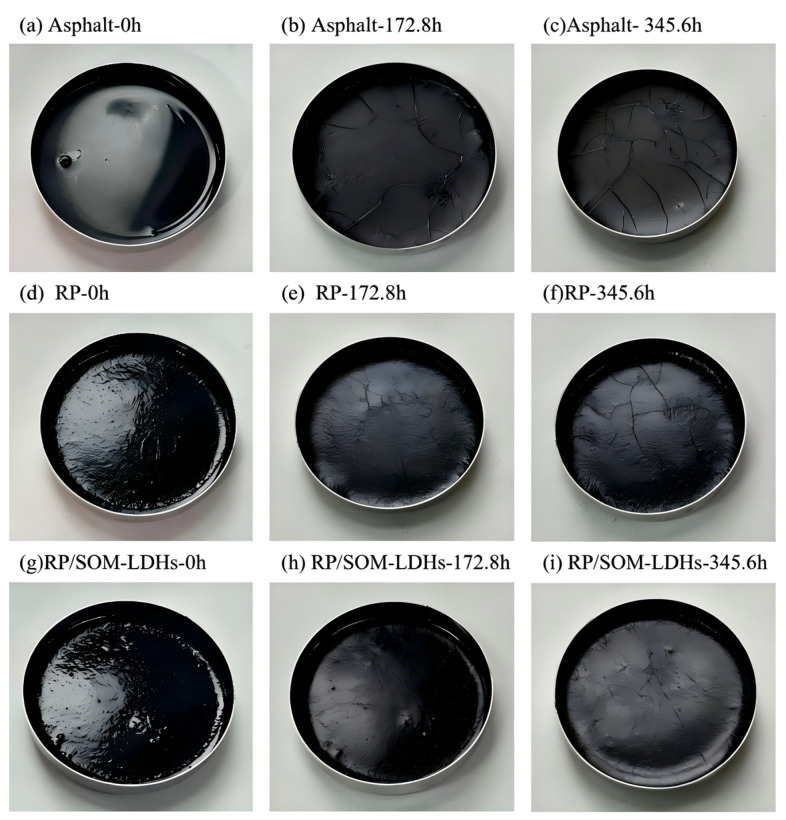
Aging surface map of different types of modified asphalt.

Mass loss before and after thermo-oxidative aging, the residual needle penetration ratio, and the viscosity ratio were determined by testing. Table 3 and Figure 4 show the key data obtained from the test.

Synthesizing the results of the previous analysis, the inclusion of rubber powder leads to a decrease in asphalt mass loss, an increase in the residual needle penetration ratio, and a decrease in the viscosity ratio after aging. When rubber powder and SOM-LDHs work together, the mass loss rate and viscosity ratio of asphalt showed a downward trend, while its residual penetration ratio increased significantly. Accordingly, the composite addition of RP and SOM-LDHs significantly inhibited the aging rate of asphalt and endowed RP/SOM-LDHs modified asphalt with outstanding anti-aging performance.

#### 2.2.2. Accelerated UV Aging Test Results

Figure 4 shows the surface morphology changes of 3 mm thick asphalt sheets prepared by asphalt after aging for 0 h, 172.8 h, and 345.6 h in a UV aging simulation test. As shown in the figure, with the extension of aging time, the three asphalt samples showed significant appearance changes.

As can be seen in Figure 4a,d,g, after undergoing UV aging for 172.8 h, all three kinds of asphalts showed an initial appearance deterioration characterization. The original smooth mirror-like surface of the matrix asphalt gloss began to decrease, the color from the initial black gradually transitioned to dark brown, and cracks began to appear. At this stage, the difference between the three kinds of asphalt was not significant; gloss and surface flatness slightly decreased, and this was accompanied by fine cracks. When the UV aging time is extended to 345.6 h, Figure 4c,f,i shows that after 345.6 h of UV aging, the color of the matrix asphalt has deepened to dark brown, indicating that the photo-oxidation reaction continues and the degree of aggravation of the surface gloss significantly weakens, tending to be close to the matte state; there is a more obvious cracking phenomenon: the cracks are linearly distributed, and they are wider and shallower. After 345.6 h of UV aging treatment, the surface crack density of RP-modified asphalt increased significantly, accompanied by crack propagation and wrinkle aggravation. The appearance deterioration trend of RP/SOM-LDHs composite modified asphalt is similar to that of RP-modified asphalt. However, the introduction of SOM-LDHs significantly improves its surface damage characteristics: the crack dispersion is more uniform, and the crack width is significantly lower than that of matrix asphalt and rubber powder modified asphalt. SOM-LDHs effectively inhibit the aging damage of asphalt caused by ultraviolet radiation.

#### 2.2.3. Dynamic Shear Rheology Test Results

(1) Temperature scanning test analysis

**Figure 5:**
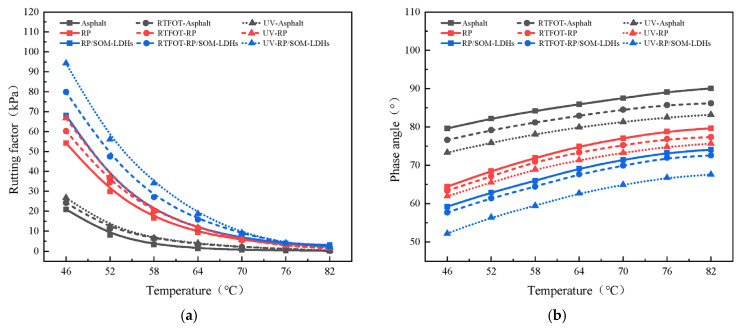
Temperature scanning test results before and after asphalt aging.

Three rheological parameters, G*, *δ,* and G*/sin δ, were obtained from the tests, and the test results of the rheological parameters of the matrix asphalt before and after the rotating film oven test (RTFOT) for ultraviolet (UV) aging, RP-modified asphalt, and RP/SOM-LDHs composite modified asphalt are shown in Figure 5.

Figure 5a shows that the G * of RP/SOM-LDHs composite modified asphalt is significantly higher than that of matrix asphalt and RP-modified asphalt at the same test temperature. The significant increase in G * value confirms that the composite modified asphalt has excellent shear deformation resistance in the medium temperature range, and can more effectively resist external shear stress, such as a driving load. After short-term aging of RTFOT, the G* values of the three asphalts increased, reflecting an improvement in high-temperature rheological properties after aging. Based on the comprehensive test data, compared with the non-aging state, the G* of RP-modified asphalt increased by 20.46%, and the G* of RP/SOM-LDHs composite modified asphalt increased by 27.35%. The above data show that the composite modification system of RP and SOM-LDHs shows a stronger enhancement effect than single RP modification.

According to the test results shown in Figure 5b, the phase angle (*δ*) of the three bitumens increases with the increase in temperature. This phenomenon indicates that the asphalt material gradually softens under high temperature environments, and the proportion of its elastic response decreases, resulting in an increase in irreversible deformation. After RTFOT aging, the *δ* values of the three kinds of asphalt decreased, which reflects the better synchronization between stress and strain responses, and a stronger elasticity recovery ability. This allows the material to recover to its original state more rapidly after bearing external force and reduces the generation of permanent deformation. After UV aging, the *δ* of RP-modified asphalt and RP/SOM-LDHs composite-modified asphalt decreased by 3.88% and 11.83%, respectively, compared with that of unaged asphalt, and 2.37% and 9.53%. This indicates that even in the face of structural changes in asphalt due to aging, the effect of RP and SOM-LDHs can further optimize the structure of asphalt so that it can better resist the deformation brought about by aging and external load.

**Figure 6:**
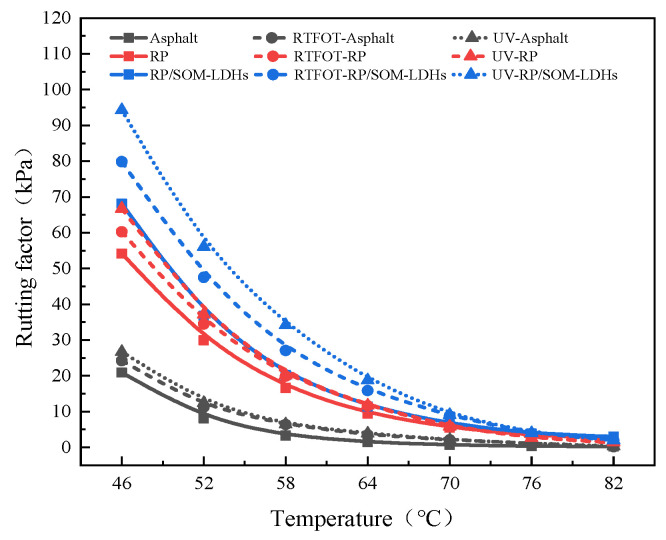
G*/sin δ-Temperature curve before and after asphalt aging.

Figure 6 shows the evolution law of rutting coefficient of asphalt materials with the increase in temperature before and after aging treatment.

As shown in Figure 6, over the test temperature range of 46 °C to 82 °C, the rutting factor (G */sin δ) of the three non-aging asphalts decreased significantly with increasing temperature. This phenomenon indicates that the material’s ability to resist rutting deformation decreases with increasing temperature. When the temperature approaches 82 °C, the difference in G */sin δ values of the three asphalt decreases, and the rate of change of the curve decreases and gradually stabilizes. In the same temperature rise process (46 °C → 82 °C), the G/sin δ value of asphalt was improved after RP modification, indicating that the poor high temperature performance of the matrix asphalt is improved by the effects of RP and SOM-LDHs, resulting in a more excellent resistance to high temperatures, which can significantly reduce the generation of rutting deformation in the actual use process. After RTFOT and UV aging, the G*/sin δ of RP/SOM-LDHs composite asphalt is always the largest, which indicates that this composite modification can effectively improve the high-temperature performance of asphalt. Taking 46 °C as an example, its G*/sin δ is both 79.86 kPa after short-term aging. This is because RP and SOM-LDHs play a modification role in asphalt, so that the mechanical strength of asphalt has been improved as a whole.

(2) Frequency sweep test analysis

**Figure 7:**
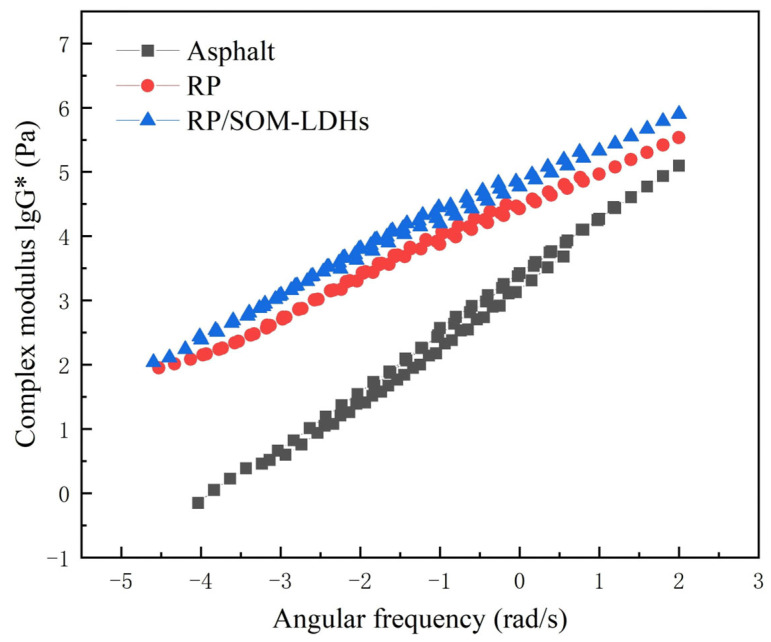
Three asphalts G*-angular frequency main curves.

The complex shear modulus (G) and angular frequency (ω) are in the form of double logarithms. By fitting the curves at different temperatures, the displacement factors of each temperature relative to the reference temperature are calculated. The test data of asphalt at each temperature were horizontally shifted according to their corresponding displacement factors. The lgG* -lg*ω* curves at other temperatures were shifted to the reference temperature (40 °C) according to the calculated logα T value along the frequency axis (lgω axis). Finally, through the above horizontal shift operation, a single master curve (lgG* vs. lg*ω*) covering a wide equivalent frequency range is obtained. Figure 7 shows the viscoelastic master curves of asphalt at the reference temperature of 40 °C.

Based on the analysis results of the main curve in Figure 7, the complex shear modulus (G*) of the matrix asphalt is at a low level under high temperature and low frequency conditions (equivalent to the actual high temperature and slow load of the pavement). This shows that when the matrix asphalt is exposed to a high temperature environment, its viscoelastic response is significantly weakened, and it is difficult to effectively resist the irreversible deformation caused by external load, reflecting its lack of high temperature resistance to permanent deformation. Comparative analysis showed that the G* values of RP-modified asphalt and RP/SOM-LDHs composite modified asphalt were significantly higher than those of matrix asphalt in the same high temperature and low frequency range. This phenomenon confirms that the introduction of RP and SOM-LDHs can effectively enhance the high temperature deformation resistance of asphalt materials. The addition of SOM-LDHs enhances the stability of the asphalt’s internal structure and shear resistance, which enables the asphalt to provide more effective resistance to deformation under shear, and thus significantly improve the overall performance of the asphalt. The G* value of RP/SOM-LDHs composite modified asphalt increases mildly at low temperature. This indicates that the internal structure of the composite modified asphalt in response to temperature changes is more stable and its temperature sensitivity has been significantly improved, making it better able to adapt to the impact of temperature fluctuations. RP/SOM-LDHs composite modified asphalt showed the highest G* value., and with the change in the conditions it showed a continuously increasing trend. This shows that RP/SOM-LDHs composite modified asphalt not only has excellent high-temperature resistance and can maintain a stable structure and performance in a high-temperature environment, but it also has excellent performance in resistance to deformation, in that it can resist the deformation caused by external forces.

#### 2.2.4. Low Temperature Bending Rheology Test Results

**Figure 8:**
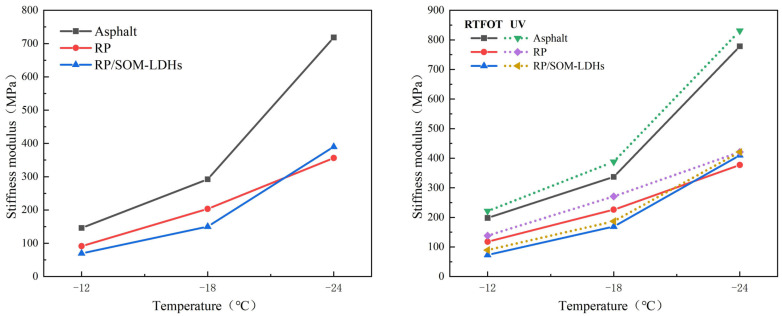
Creep modulus of asphalt at different temperatures before and after aging.

**Figure 9:**
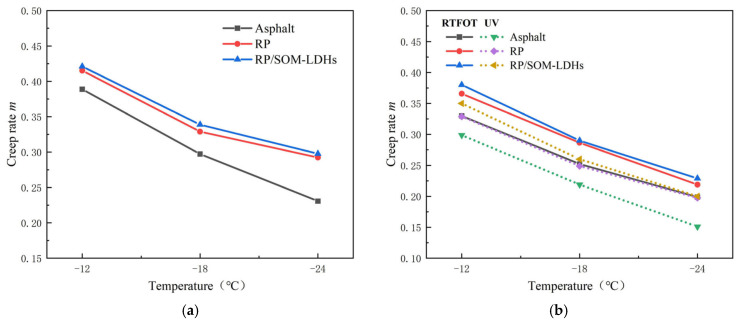
Asphalt creep rate at different temperatures before and after aging.

Figure 8 and Figure 9 show the test results of creep stiffness and creep rate of asphalt, respectively.

The results shown in Figure 8 indicate that the creep stiffness S (MPa) of the three types of asphalt, both before and after aging, increases as the temperature decreases. The S values of RP-modified asphalt and RP/SOM-LDHs composite modified asphalt show a significant decreasing trend compared with base asphalt. It can be seen that the composite modification effect of RP and SOM-LDHs at −12 °C and −18 °C is better than that of RP alone. At −24 °C, the single modification with RP is more conducive to improving the low-temperature crack resistance of asphalt. This is because after rubber powder is mixed with the asphalt matrix, its particles can absorb and dissipate part of the stress through physical entanglement and deformation; it also plays a role in low-temperature plasticizing. The elastic properties of rubber powder give asphalt a certain elastic deformation capacity when subjected to stress, thus enhancing the low-temperature ductility of asphalt. After UV and RTFOT aging, the S values of all three asphalts increased compared with those before aging. The S values of RP-modified asphalt and composite modified asphalt remain lower than those of base asphalt, indicating that the modification effect of RP and SOM-LDHs remains stable after aging. At −24 °C, the effect of RP modification alone is even more evident, which is consistent with the pre-aging performance. The elastic properties of rubber powder enable the asphalt to maintain a certain degree of flexibility at this very low temperature, and the composite modified asphalt, although not showing better low-temperature performance than the RP-modified asphalt at −24 °C, still maintains a certain level of resistance to low-temperature deformation.

Based on the test results shown in Figure 9a, at the same test temperature, the m-value (creep rate) of the RP/SOM-LDHs composite modified asphalt is higher than that of base asphalt and RP-modified asphalt. The introduction of the composite modifier effectively improves the low-temperature brittleness of asphalt and makes it more plastically deformable at low temperatures, thereby effectively alleviating stress concentration and reducing the risk of low-temperature cracking. In the low-temperature environment, the effect of RP and SOM-LDHs significantly improves the low-temperature plastic deformation ability of asphalt and endows it with more excellent stress relaxation characteristics, which is the core mechanism for the enhancement of low-temperature crack resistance of the composite modified asphalt. According to Figure 9b, the m-values of the three asphalts decrease after RTFOT and UV aging, indicating that molecular movement becomes more difficult and the deformation capacity of the material decreases, leading to poorer low-temperature performance. The incorporation of RP and SOM-LDHs slows down the magnitude of change in the creep rate of asphalt. This is because rubber powder has good elasticity and flexibility; it can absorb and disperse stress in the asphalt and prevent or delay the crosslinking and hardening of asphalt molecules during aging, thus making it less prone to low-temperature shrinkage and cracking.

#### 2.2.5. Multi-Stress Creep Recovery Test Results

**Figure 10:**
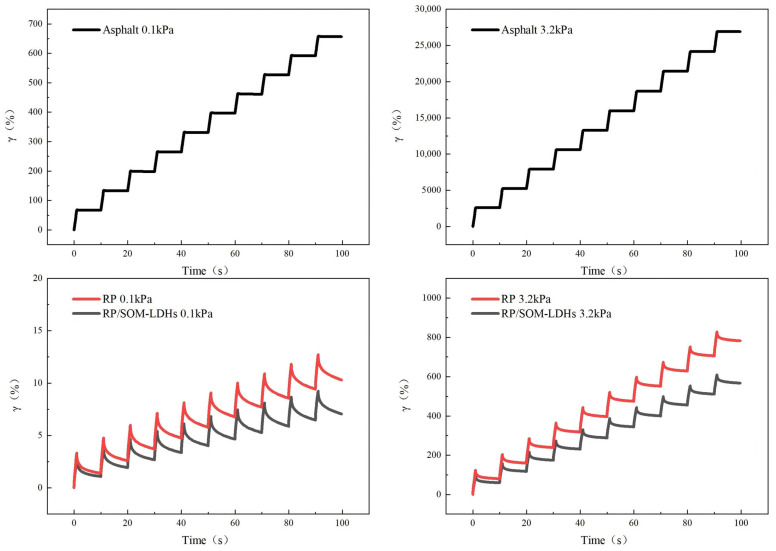
MSCR test data diagram.

The MSCR test temperature in this section is 64 °C, and the specimen size is 25 mm diameter × 2 mm thickness with 2 mm parallel plate gap; the test results are shown in Figure 10.

From Figure 10, under the high temperature condition of 64 °C, when matrix asphalt is subjected to 0.1 kPa and 3.2 kPa stress loading, the change curves of strain (γ) versus time (t) in each creep cycle are nearly at right angles. The test results indicate that the matrix asphalt encounters significant shear deformation, and after stress unloading, the value of irrecoverable strain is large and the strain recovery capacity is seriously insufficient. The observations of Figure 10 can be seen in the full creep cycle under the same test conditions, and the degree of unrecoverable strain of the matrix asphalt compared to the modified asphalt is significantly larger. Under high ambient temperature conditions, matrix asphalt shows a significant disadvantage in terms of suppressing the deformation tendency and maintaining its own morphological stability. The incorporation of RP strengthens matrix asphalt elastic recovery performance, and improves the asphalt morphology stability. Performance has been strengthened to enhance the creep recovery ability, SOM-LDHs blending based on the existing performance of RP-modified asphalt further optimizes road engineering construction, providing a reference base for screening and application of high-performance asphalt materials.

### 2.3. Microscopic Mechanism Analysis

#### 2.3.1. Micro-Morphological Analysis of Composite Modified Bitumen

(1) Analysis of scanning electron microscope test results

**Figure 11:**
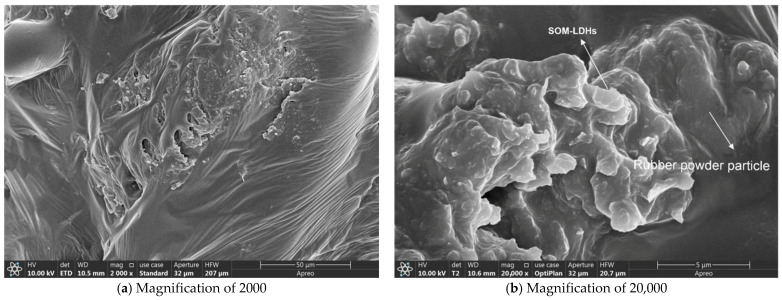
Scanning electron microscopy image of RP/SOM-LDHs composite modified asphalt.

Scanning electron microscopy (SEM) analysis of the RP/SOM-LDHs composite modified asphalt was performed at a magnification of 2000 and 20,000 times, and its micro-morphology is shown in Figure 11.

The SEM images reveal that SOM-LDHs nanoparticles are uniformly dispersed on the rough surface of rubber powder particles, acting as nanoscale crosslinkers that bridge the rubber phase and asphalt matrix. The inorganic nanoparticles enhance the stability of the organic elastic phase, thereby improving the structural integrity and thermal stability of the composites. In the micro-morphology of RP/SOM-LDHs composite modified asphalt under 2000 times magnification, the distribution of rubber powder particles within the asphalt matrix is not highly homogeneous, and there is a localized relative aggregation phenomenon even though large-scale agglomerates have not been formed, which may be related to the distribution of the shear force in the process of composite modification as well as interactions between the components. Since the SOM-LDHs are nanomaterials, it is difficult to observe the specific fusion details under 2000 times magnification. After increasing the magnification to 20,000 times, it can be observed that the surface of the rubber powder particles is highly rough and is covered with a large number of tiny concave and convex structures and pores, which provide abundant sites for physical adsorption, entanglement, and chemical cross-linking with the asphalt and SOM-LDHs. The SOM-LDHs exist in nanometer or submicron particle form, either attached to the surface of the rubber powder particles or dispersed in the asphalt matrix. This phenomenon indicates that there is no obvious repulsion between SOM-LDHs and rubber powder and asphalt in the composite modification process, and that the bonding state is good.

(2) Analysis of Atomic Force Microscopy Test Results

**Figure 12:**
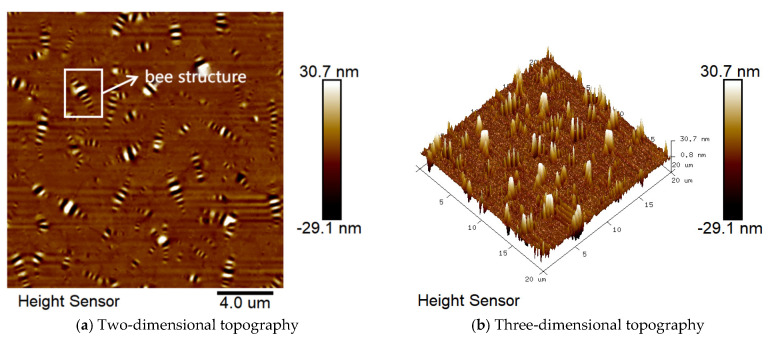
Microscopic morphology of matrix asphalt.

**Figure 13:**
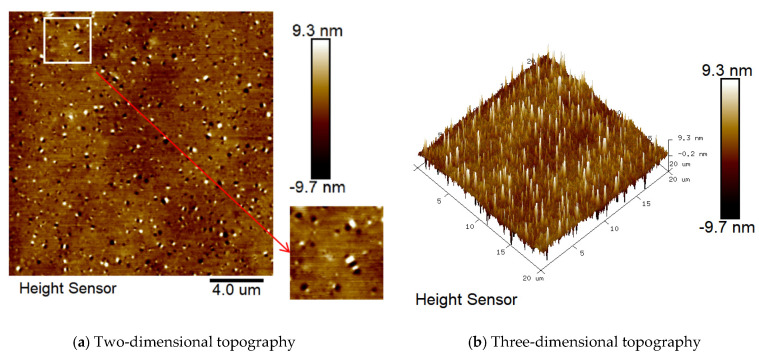
Microscopic morphology of RP-modified asphalt.

**Figure 14:**
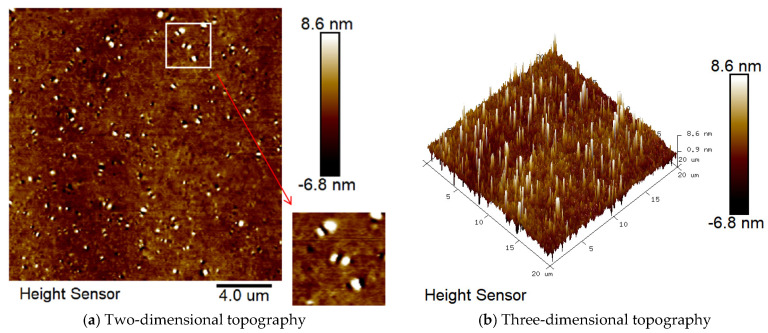
Microscopic morphology of RP/SAM - LDH composite modified asphalt.

Figure 12, Figure 13 and Figure 14 show the microstructure characteristics of asphalt.

**Figure 15:**
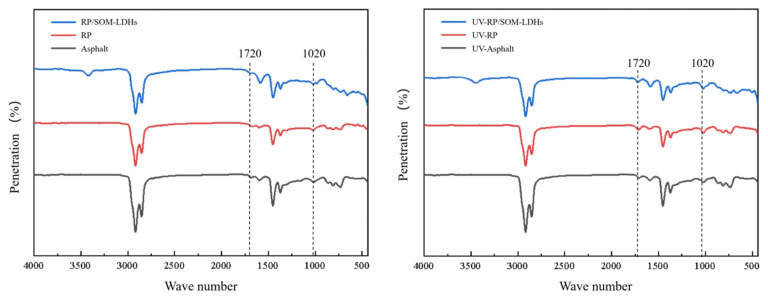
Infrared spectra of asphalt before and after aging.

Based on the microstructure characterization results of matrix asphalt shown in Figure 12, it can be observed that the honeycomb structure characteristics of multi-scale distribution are uniformly dispersed inside. In the “bee-shaped structure”, wax crystals act as the nucleation center; with the decrease in temperature, the other components around them agglomerate. Due to the different mechanical characteristics of the agglomerate and matrix phase, the combination of curvature elastic strain helps maintain the system energy constant, so the wax crystals form a corrugated structure to offset strain, thus leading to the formation of the “bee-shaped structure” along the long-axis direction [24]. It is also believed that the bee-shaped structure is wax molecules, asphaltene, and other macromolecules, such as long-chain alkyl eutectic formation of wax crystallization conclusions [25]. Its homogeneous dispersion indicates the existence of stable and relatively homogeneous interactions between wax, asphaltene, and colloidal and other components of asphalt. There is a clear correspondence between the color and the structural morphology in the 2D morphology, in which the white part corresponds to the raised area in the asphalt structure at the microscopic level and to the wave crest in the 3D map, and the dark part corresponds to the concave area in the 3D map. The microscopic morphology of the RP-modified asphalt in Figure 14 shows a white dot-like structure, which, upon magnification, is found to be a reduced honeycomb structure; at the same time, the degree of surface protrusion is significantly reduced with respect to the base asphalt. The addition of the rubber powder to the asphalt absorbs the lighter components of the asphalt matrix and the asphaltene increases, which in turn leads to the reduction in the honeycomb structure [26]. The roughness reduction of RP-modified asphalt can also be understood as a result of the reduced bee-shaped structure in RP-modified asphalt. Figure 15, in the RP/SOM-LDHs composite modified asphalt micro-morphology, shows a white dotted structure similar to that in the RP-modified asphalt. When zoomed in, the honeycomb structure is reduced, and the surface roughness is also greatly reduced. Overall, the performance is roughly the same as that of RP-modified asphalt. From a comprehensive comparison of the three kinds of asphalt microscopic morphologies, it can be concluded that the rubber powder is the main factor making the asphalt internal honeycomb structure smaller, due to the SOM-LDHs’ nanoscale materials, small particle size, and low dosage. To the naked eye, it is difficult to observe the changes in morphology in the graph; it needs to be combined with the surface roughness indicators to analyze the mechanism of its role.

In order to deeply and accurately analyze the differences between the three kinds of asphalt in terms of microscopic surface morphology from a quantitative point of view, this section applies the two indexes of Rq and Ra for analysis. The calculation results are shown in Table 4.

R_q_ indicates that the asphalt surface microscopic degree of undulation is more significant; the surface is more rough and irregular. The smaller value indicates that the asphalt surface is smooth. R_a_ being larger indicates that the asphalt surface contour deviates from the center line of the average degree; that is, the surface roughness is higher. The smaller value indicates that the asphalt surface is relatively more flat, and that the average surface contour of the undulation is smaller. From the test results shown in Table 4, there are three kinds of asphalt R_a_. From largest to smallest, these are matrix asphalt > RP-modified asphalt > RP/SOM-LDHs composite-modified asphalt, of which the R_a_ of RP-modified asphalt decreased by 55.48% and the R_a_ of RP/SOM-LDHs asphalt decreased by 60.42% compared with matrix asphalt. This indicates that both RP modification and RP/SOM-LDHs composite modification reduce the roughness of the asphalt surface. From the effect on asphalt microscopic roughness, the rubber powder played a major role in reducing the asphalt surface roughness, which is consistent with the conclusion of the microscopic morphology map derived from the test. Rubber powder absorbs the lightweight components of the asphalt to form a swelling structure, affecting the formation of asphalt in the “bee structure”, so that the rubber-modified asphalt microscopic roughness is significantly smaller than the base asphalt. SOM-LDHs are added in the existing modified basis for further optimization, and SOM-LDHs are added to RP-modified asphalt; its unique layered structure can be combined with the rubber asphalt, and its unique layered structure can be combined with the rubber asphalt. The unique laminated structure can cooperate with the swelling structure of the rubber powder to further fill the voids inside the asphalt, prompting the asphalt molecules to be arranged more orderly and closely, thus further reducing the surface roughness of RP/SOM-LDHs composite modified asphalt.

#### 2.3.2. Analysis of Aging Mechanism of Composite Modified Asphalt

(1) Analysis of infrared spectroscopy test results

Figure 15 shows the Fourier transform infrared (FTIR) spectra of asphalt before and after UV aging. The integral analysis of the characteristic absorption peak area was carried out by OMNIC software. T 9.2he calculati5 uoculation results are summarized in Table 5.

Based on the FTIR spectrum analysis of asphalt before and after UV aging shown in Figure 15, the strong dipole moment and large vibration amplitude of the antisymmetric methylene group indicate that the matrix bitumen contains saturated hydrocarbons. At about 1600 cm^−1^, there is a characteristic peak band of low transmittance, belonging to the absorption peaks of aromatic compounds. At 1450 cm^−1^ and 1380 cm^−1^, the stretching vibration peaks originated from the symmetric surface vibration in the methylene (-CH_2_-) plane and the symmetric variable angle vibration of the methyl functional group (-CH_3_-), which indicates that the asphaltene molecule contained a saturated hydrocarbon structure. At 1020 cm^−1^, the absorption peak is weak. Between 710 cm^−1^ and 879 cm^−1^, there is a weak absorption peak, corresponding to the benzene ring substitution region, caused by the out-of-plane rocking vibration of the C-H group on the benzene ring and the vibration of the C=C skeleton, which suggests the presence of aromatic compounds in the matrix asphalt. The spectra of RP-modified asphalt do not show new characteristic absorption peaks compared to that of matrix asphalt, and this phenomenon suggests that there are no new characteristic absorption peaks in the RP-modified asphalt during the process of RP modification. This phenomenon shows that in the preparation of RP-modified asphalt, rubber powder and matrix asphalt did not undergo chemical reactions; they only interacted via physical mixing.

As can be seen from Table 5, the carbonyl and sulfoxide functional group indices of the three asphalts were basically the same before aging, and when the three asphalts underwent the thermo-oxidative and UV aging process, these free radicals rapidly reacted with oxygen in an aerobic environment in an oxidative reaction. Under UV radiation, the chemical bonds in the asphalt molecules absorb energy and break, which in turn generates highly reactive free radicals. The rate of change of *I_C=O_* and *I_S=O_* before and after aging of matrix asphalt reached 7.47203 and 0.64279, respectively, which indicates that in the process of UV aging of the matrix asphalt, its internal chemical structure has undergone more drastic changes, and the ability to resist UV aging is relatively weak. This change indicates that the addition of rubber powder improves the aging resistance of asphalt, which is due to the fact that the rubber powder can form a network structure in the asphalt system, which can play a certain role in blocking the UV light. For the RP/SOM-LDHs composite modified asphalt, the rate of change of *I_C=O_* and *I_S=O_* before and after aging is more obviously reduced. This is mainly because the lamellar structure of SOM-LDHs can shield part of the ultraviolet light and impede the penetration and diffusion of oxygen into the asphalt, making it difficult for oxygen to fully contact with the asphalt molecules to oxidize. Therefore, the addition of SOM-LDHs effectively inhibits the photo-oxidative UV aging process of asphalt and significantly reduces the destructive effect of UV light on asphalt, thereby greatly improving the UV aging resistance of asphalt.

(2) Analysis of the results of the gel permeation chromatography test

**Figure 16:**
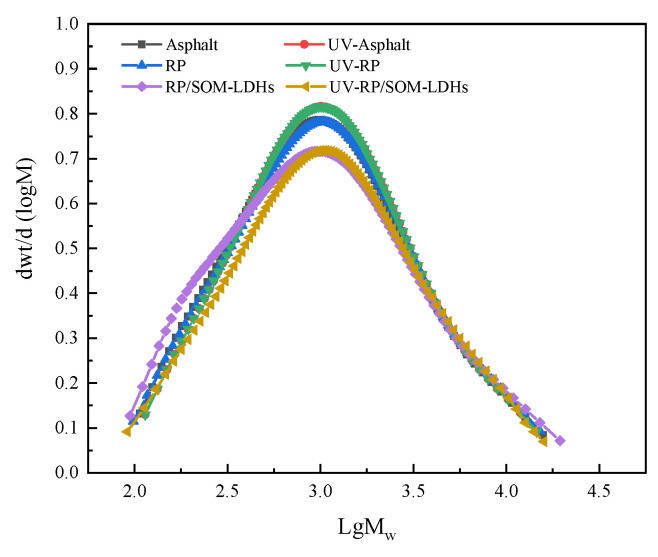
Molecular weight distribution of different types of asphalt.

Figure 16 shows the evolution of molecular weight distribution of asphalt before and after UV aging.

In Figure 4, Figure 5, Figure 6, Figure 7, Figure 16, (X axis) represents the logarithm of weight-average molecular weight (Mw) (lg Mw), and the ordinate (Y axis) represents the relative content of asphalt components. It can be seen from the analysis map that as the aging degree increases, the molecular weight distribution curve of asphalt migrates to the high molecular weight region, indicating that the relative content of macromolecular components increases. The weight-average molecular weight (Mw), number-average molecular weight (Mn), and molecular weight distribution index (PDI) of three kinds of asphalt before and after aging were calculated. The specific results are listed in Table 6.

Based on the analysis of the data in Table 6, the introduction of the modifier significantly changed the weight-average molecular weight (Mw) of the asphalt. The test results confirmed that the composite addition of rubber powder (RP) and SOM intercalated LDHs (SOM-LDHs) effectively enhanced the deformation resistance of the asphalt matrix and significantly contributed to improving its high temperature stability.

The change trend of the molecular weight distribution index (PDI) of base asphalt, RP-modified asphalt and RP/SOM-LDHs composite modified asphalt is consistent with the change rule of their weight-average molecular weight (Mw). The PDI values of these three asphalts are ranked as follows: RP/SOM-LDHs composite modified asphalt is larger than RP-modified asphalt is larger than base asphalt. PDI is a key quantitative indicator to characterize the polydispersity of molecular weight. When the PDI value is low, the molecular weight distribution is more uniform, and the proportion of components in a specific molecular weight range is higher. It is worth noting that the wider molecular weight distribution is conducive to improving the asphalt system. With a high temperature stability, RP/SOM-LDHs composite modified asphalt presents the widest molecular weight distribution.

After UV aging, the *M_w_* values of all three asphalts showed an increasing trend, and comparing growth rates of their *M_w_* changes, it can be found that the different asphalts underwent different degrees of aging and the *M_w_* growth rate of the asphalt samples decreased with the addition of RP and SOM-LDHs. Due to the rubber powder providing elasticity and flexibility, its molecular structure contains many unsaturated bonds and reactive groups; in the UV aging process, rubber powder can reduce the light energy directly absorbed by the asphalt molecules, which reduces the photochemical reaction rate of the asphalt molecules. Rubber powder and asphalt molecules physically limit the movement and reactivity of the asphalt molecules, so that the degree of intermolecular crosslinking and polymerization is low, resulting int a decrease in the growth rate of *M_w_*. Therefore, the degree of aging is relatively reduced. The lowest *M_w_* growth rate of RP/SOM-LDHs composite modified asphalt is attributed to the improvement in the anti-aging properties of asphalt due to the joint action of SOM-LDHs and RP/SOM-LDHs having a special lamellar structure and a large specific surface area. The organization of its interlayers provides better compatibility with asphalt molecules. The layered structure of SOM-LDHs can play the role of a physical barrier, blocking the penetration of ultraviolet rays and reducing radiation damage to the asphalt molecules. Together with the above anti-aging effects of rubber powder, the composite of the two resulted in the improved anti-aging properties of the asphalt, with a further reduction in the *M_w_* growth rate and the lightest degree of aging.

## 3. Conclusions

(1) RP dosage, SOM-LDHs dosage, and shear temperature were selected as the influencing factors of the response surface method, and 17 groups of experiments were designed using the BBD response surface method, with the degree of penetration, ductility, and softening point as the response indexes; the optimal preparation scheme of RP/SOM-LDHs composite modified asphalt was finally determined to be as follows: RP dosage of 21.7%, SOM-LDHs dosage of 4.8%, and shear temperature 160 °C.
(2) Compared with matrix asphalt, the high-temperature performance of RP/SOM-LDHs composite modified asphalt is significantly improved. After RTFOT and UV aging, the complex shear modulus G increased by 27.35%, and the rutting factor G/sin δ reached 79.86 kPa at 46 °C. The phase angle δ decreased by 11.83% after UV aging, indicating that it had excellent resistance to permanent deformation.
(3) In the low temperature range of −18 °C to −24 °C, compared with the matrix asphalt, the creep stiffness S of the composite modified asphalt is reduced by about 30%, and the m value is increased by about 15%. The strain recovery rate R at 3.2 kPa stress is 78.5%, and the unrecoverable creep compliance Jnr is as low as 0.18 kPa^−1^. These results indicate that the effect of RP and SOM-LDHs significantly improves the low temperature crack resistance and elastic recovery ability of asphalt.
(4) Microstructure characterization showed that SOM-LDH nanoparticles were uniformly dispersed on the rough surface of rubber powder particles and acted as physical crosslinking nodes. Compared with the matrix asphalt, the surface roughness of the composite modified asphalt is reduced by 60.42%. The lowest growth rate of the aging index was IC = O and IS = O (3.71201 and 0.45314, respectively), and the growth rate of Mw was only 19.27%, which confirmed the excellent anti-aging performance of SOM-LDHs’ layered structure.

This study successfully developed RP/SOM-LDHs composite modified asphalt, which improved high-temperature deformation resistance, low-temperature crack resistance, and UV aging resistance. However, limitations include insufficient characterization of the dynamic chemical processes (e.g., particle size evolution, critical gel point, and crosslinking mechanism) and the lack of SOM-LDHs-only and untreated-LDHs controls, which prevented the quantitative separation of individual contributions. Future work will employ dynamic light scattering, rheological time sweeps, and in-situ microscopy to dynamically track sol-gel transformation; combine molecular simulation and interfacial analysis to establish quantitative structure–activity relationships; perform FTIR peak deconvolution for accurate oxidation indices; and link GPC-derived molecular weight evolution with rheology to understand anti-aging performance. The influence of shear temperature (160, 175, 190 °C) will be systematically evaluated to determine the optimal processing window. A full factorial design including base asphalt, RP alone, SOM-LDHs alone, composite, and KH560-only/untreated LDHs controls will be implemented to quantitatively evaluate individual and interactive effects on network formation and performance enhancement.

## 4. Materials and Methods

### 4.1. Raw Materials

#### 4.1.1. Base Asphalt

GradeA70# base asphalt used in this study was purchased from Zhengzhou Municipal Engineering Corporation. As per “Test in Henan Province Procedures for Asphalt and Asphalt Mixture in Highway Engineering” (JTG E20-2011) [22], the basic performance test of matrix asphalt was carried out; at the same time, the technical indexes were evaluated with reference to “Technical Specifications for the Construction of Highway Asphalt Pavements” (JTG F40-2004) [27], and the relevant results are listed in Table 7.

#### 4.1.2. LDHs

The LDHs (Mg_4_Al_2_(OH)_12_CO_3_·4H_2_O) selected for this study were provided by Tianjin Jinheng Blue Ocean Science and Technology Co. Ltd. with the appearance of white powder. Table 8 summarizes the key physical performance parameters of related materials.

**Table 8:** Basic physical properties of LDHs.

Magnesium Aluminum Ratio (Molar Ratio)	Whiteness Wr	Moisture (105 °C, w%)	Mean Diameter (D50)	PH (1 g/100 mI H_2_O)
2.25	98	0.56	<6 μm	9.8

#### 4.1.3. Rubber Powder

The rubber powder used in this study was supplied by Hebei Kexu Building Materials Co., Ltd. It is produced from waste truck tires by ambient grinding and has a nominal mesh size of 40 mesh (0.425 mm opening). According to the supplier’s specification, the particle size distribution of the rubber powder is as follows: 100% passes through a 0.595 mm (30 mesh) sieve, at least 90% passes through a 0.425 mm (40 mesh) sieve, and approximately 70% passes through a 0.250 mm (60 mesh) sieve. Other physical properties of the rubber powder are listed in Table 9.

#### 4.1.4. Coupling Agent

KH560 is an aminosilane coupling agent with good reactivity and adhesion properties. Its molecule contains amino groups that chemically react with many organic and inorganic materials to enhance bonding and durability. KH560 is commonly used to modify polymers, enhance the performance of composites, and improve the adhesion of coatings.

#### 4.1.5. Surface Modification of LDHs

The coupling agent used is KH560 (γ-glycidoxypropyltrimethoxysilane), an epoxy-functional silane with the molecular formula C_9_H_20_O_5_Si. It contains three methoxy groups (–OCH_3_) for hydrolysis and condensation with hydroxyl groups on LDHs surfaces, and a terminal epoxy group for reaction with polar components of asphalt. In this study, KH560 acts as a molecular bridge: the methoxy groups form covalent Si–O–M bonds with LDHs, while the epoxy group interacts with asphalt matrix.

### 4.2. Preparation and Formulation Optimization of Modified Asphalt

#### 4.2.1. Preparation of Surface Organicized LDHs

**Figure 17:**
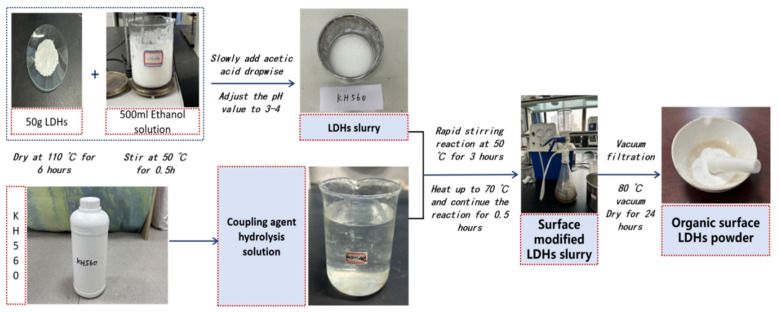
Preparation process of surface-organic LDHs.

In this study, KH560 was selected to modify LDHs by surface organicization, and surface organicized LDHs were prepared. The preparation process of surface organicized LDHs is shown in Figure 17, and the experimental steps are as follows:

#### 4.2.2. Preparation of RP/SOM-LDHs Composite Modified Asphalt

(1) RP-modified asphalt preparation

The preparation process of RP-modified asphalt mainly includes three key links: swelling, high-speed shearing, and refining. First, the weighed matrix asphalt was heated in an oven at 180 °C to enhance its fluidity and promote interaction with rubber powder. Subsequently, 20% mass fraction of 40-mesh fine rubber powder was slowly added to the hot bitumen [28]. The rubber powder was maintained in the asphalt for a dissolution time of 2 h to ensure that it swells sufficiently and forms an effective interfacial bond with the asphalt matrix. After completion of swelling, the mixture was transferred to a high-speed shear. Fine dispersion of rubber particles was achieved by continuous shearing at 4000 r/min for 60 min at a constant temperature of 190 °C [29]. The sheared material was left to develop under set conditions for 45 min. This processing method can strengthen the physical entanglement of rubber particles and may trigger potential chemical crosslinking, thereby improving the mechanical properties and long-term service performance of modified asphalt. Finally, RP-modified asphalt samples with different rubber powder contents were successfully prepared by this process.

(2) Preparation of RP/SOM-LDHs composite modified asphalt preparation

First, the matrix asphalt is heated to 175 °C; this temperature can not only effectively reduce the viscosity of asphalt, but also promote the movement of its molecular chain, so that it is easier to interact with the additives in the subsequent modification process. According to the design scheme determined in the response surface optimization method, different dosages of rubber powder and a certain amount of SOM-LDHs were added slowly and uniformly, and the mixture was allowed to fully dissolve for 2 h to ensure that the rubber powder and SOM-LDHs formed a homogeneous dispersion system. Subsequently, the mixture was subjected to continuous shearing for 60 min by a high-speed shear, with the rotational speed set at 4000 r/min and the development time of 45 min. Thus, the RP/SOM-LDHs composite modified asphalt was obtained. This process not only helps to further break the aggregates of the rubber powder and promote its homogeneous dispersion in the asphalt matrix, but also improves the dispersion of SOM-LDHs, which enhances the overall performance of the composite. During the preparation process, the SOM-LDHs, with their organic surface functional groups, interact with the polar components of asphalt through hydrogen bonding and physical adsorption, forming localized crosslinking sites. Simultaneously, the rubber powder absorbs light components and swells, establishing a continuous elastic phase.

#### 4.2.3. Response Surface Methodology to Optimize the Experimental Design Scheme

In this paper, the factors and interactions between factors were analyzed by the Box–Behnken design response surface method (BBD) using the experimental analysis design software Design-expert (DE8, version 13). Based on the above analysis, the experimental design variables were A (RP doping 18%~22%), B (SOM-LDHs doping 2%~6%), and C (shear temperature 160 °C~190 °C), and the needle penetration (25 °C, 100 g, 5 s), softening point, and elongation (5 °C, 50 mm/min) were used as the response indexes [30,31,32]; the response surface methodology model was carried out under the BBD response surface method. A composite experimental design was used to establish the response surface model. The validity and predictive power of the model needs to be assessed by its goodness of fit (R^2^ value) and significance test (*p*-value). In response surface design, different levels of the independent variable are usually selected for the experiment. To simplify the analysis, the independent variables are usually coded. A common way of coding is to standardize the independent variables to the range of [−1, 1], and in Table 1, the levels of the independent variable *X_i_* are defined as follows: low level: *X_i_, min* = −1; medium level: *X_i_, mid* = 0; and high level: *X_i_, max* = 1. The parameters of the experimental design are shown in Table 10, and the table of the experimental design scheme is shown in Table 11.

**Table 10:** Experimental design parameter table.

Factor	Name	Unit	Low Level *X_i,min_* = −1	Medium Level *X_i,mid_* = 0	High Level *X_i,max_* = 1
A	RP dosage	%	18	20	22
B	SOM-LDHs content	%	2	4	6
C	Shearing temperature	°C	160	175	190

**Table 11:** Experimental design scheme table.

Number	Test Factors		
	A: RP Dosage(%)	B: SOM-LDHs Content(%)	C: Shearing Temperature(°C)
1	20	4	175
2	18	2	175
3	20	4	175
4	22	6	175
5	20	4	175
6	20	2	190
7	20	6	160
8	18	4	160
9	20	4	175
10	20	2	160
11	20	6	190
12	18	4	190
13	18	6	175
14	22	4	190
15	20	4	175
16	22	4	160
17	22	2	175

### 4.3. Macroscopic Performance Tests

#### 4.3.1. Rotating Film Oven Test

In this study, the rotating film oven test (RTFOT) was used to simulate the thermo-oxidative aging that occurs during asphalt paving, as shown in accurately assessing the aging characteristics of asphalt under actual construction conditions, this paper strictly follows the requirements of T0610 in the protocol [22]. Samples of asphalt were accurately weighed at 35 ± 0.5 g each, respectively, and placed in special bottles for the rotating film oven (RTFOT). The sample bottles were rotated at 163 ± 0.5 °C with a rotational speed of 15 ± 0.2 r/min, while hot air at a flow rate of 4000 mL/min was passed through the bottles for 85 min of continuous aging. In order to ensure the accuracy and repeatability of the test data, all aging operations should be completed within 72 h after sample preparation.

#### 4.3.2. UV Accelerated Aging Test

Asphalt ultraviolet accelerated aging (UVA) simulation tests are designed to replicate the photo-oxidative aging behavior experienced by asphalt pavements in actual service environments. This test was performed on the RTFOT after three kinds of asphalt UV aging tests. The selected UV light source power was 100 W, the radiation area was 0.4 m^2^, the UV aging equipment UV output power was 250 W/m^2^, the UV wavelength was 365 nm, and this specific wavelength of UV light plays a key role in UV aging because it is closer to the simulation of the scientific nature and effectiveness. At the same time, the aging device is installed with an air-cooled temperature control system. In the process of asphalt UV aging, you can control the ambient temperature around the specimen so that it is maintained in a suitable and stable range to avoid interference with the test results due to temperature fluctuations, thereby ensuring that the test is accurate and repeatable.

In this study, the total annual radiation in the Tibetan Plateau region was selected to be up to 730 kJ/cm^2^-a, of which the proportion of UV radiation is about 5%, and the daily average total amount of UV radiation is calculated to be about 100 J/cm^2^·d, which is converted into the daily average power unit of 10 W/m^2^.

The power of the UV light source selected for this test is 100 W, the radiation area is 0.4 m^2^, and the UV output power of the UV aging equipment is 250 W/m^2^.

According to the principle of energy equivalence, the indoor simulation again time for one month of outdoor UV radiation is calculated using Formula (4):

(4) $$Indoor\;aging\;time=\frac{Outdoor\;aging\;power\times Outdoor\;aging\;time\;(One\;month)}{Indoor\;aging\;power}=\frac{10\;W/m^{2}\times 30\times 24\times 3600\;s}{250\;W/m^{2}}\approx 28.8\;h$$

Then the corresponding table of indoor and outdoor UV aging time is shown in Table 12.

#### 4.3.3. Dynamic Shear Rheology Test

A total of nine sets of specimens of three types of asphalt before and after undergoing RTFOT and UV aging were subjected to temperature scanning tests. The test temperature is selected between 46 °C and 82 °C., and a strain amplitude of 10% and a fixed angular frequency of 10 rad/s were used [33]. A 25 mm diameter parallel plate fixture was used, and the thickness of the asphalt specimens was controlled at 1 mm. Frequency scanning tests were conducted on the same nine groups of asphalt specimens before and after aging, as described above. The test temperature points were selected as 40 °C, 52 °C, 64 °C, 76 °C, and 88 °C, the strain amplitude was set at 1%, and the angular frequency scanning range was from 0.1 to 100 rad/s. The focus was on the variation characteristics of complex shear modulus (G*) with angular frequency (ω). Based on the frequency scanning data, the master curves of the relationship between complex modulus (lgG *) and angular frequency (lgω) of three kinds of asphalt at reference temperature were constructed. Based on the analysis of the morphological characteristics of the main curve, its evolution behavior is clarified, the modification effects of rubber powder (RP) and SOM-inserted LDHs (SOM-LDHs) on the matrix asphalt were comprehensively evaluated.

#### 4.3.4. Low Temperature Bending Rheology Test

In this study, the low temperature bending creep strength of matrix asphalt, RP-modified asphalt, and RP/SOM-LDHs composite modified asphalt was tested before and after RTFOT and UV aging treatment, which strictly follow the requirements of the Superpave specification [28]. The test temperatures were −12 °C, −18 °C, and −24 °C, respectively. During the test, continuous stress loading was applied to each specimen for a duration of 4 min to observe its deformation characteristics at low temperatures. The rheological parameters S, m were obtained from the test, and the modulus of strength S reflects the low-temperature flexibility properties of asphalt; the smaller the value is, the better the low-temperature performance of the asphalt; the creep rate m indicates the stress relaxation performance of asphalt, and the larger the value is, the more effective the asphalt is in resisting low-temperature cracking.

#### 4.3.5. Multi-Stress Creep Recovery Test

The test uses a constant loading program, repeated loading and unloading of asphalt specimens, a total of 10 creep cycles, a single creep cycle length of 10 s, and a total cycle length of 100 s [34]. This section of the test determines the creep recovery ability of asphalt specimens under two types of stress, 0.1 kPa and 3.2 kPa, under the action of the loading.

### 4.4. Microscopic Characterization and Mechanism Tests

#### 4.4.1. Scanning Electron Microscope Test

Scanning electron microscope (ThermoFisher-ApreoC) is used to characterize the micro-morphological features of the composite modified asphalt. According to the SEM selected in this paper was produced by Chinainstru & Quantumtech (Hefei) Co., Ltd. (Hefei, Anhui Province, China), the interaction mechanism between a low energy electron beam and the material surface atoms, high-resolution images are generated through the electron-optical system, and a systematic comparison of the binding state of the modifier and the asphalt, along with multiscale magnification observation, to reveal the dispersion of the external dopant and the interfacial bonding characteristics. It provides morphological evidence for the spatial distribution effect and microscopic action mechanism of the doped modifier.

#### 4.4.2. Atomic Force Microscope Test

The composite modified asphalt samples were tested with the help of AFM (Bruker Dimension Fast Scan) to derive the three-dimensional morphology and two-dimensional phase diagrams, to analyze the parameters of asphalt microstructure and microroughness, and to evaluate the microstructural characteristics of asphalt. The atomic force microscope (AFM) used in this paper is produced by Quantum Design (Beijing, China). The AFM test was performed in Tapping Mode. The key parameters were set as follows: scanning rate of 1 Hz, image acquisition resolution of 512 × 512 pixels, scanning area size of 20 μm × 20 μm, lateral resolution of 10 nm, and the temperature of the test environment was controlled at 25 °C.

#### 4.4.3. Fourier Transform Infrared Spectroscopy Test

Based on the principle of Fourier transform infrared spectroscopy test (FTIR), the molecular structure evolution of RP/SOM-LDHs composite modified asphalt before and after UV aging was systematically compared to analyze the form of interaction between the modifier and asphalt and its inhibitory mechanism on aging behavior. The samples were taken by the experimental coating method, and heated into liquid drops in the grooves on the potassium bromide wafers were tested at a scanning range of 4000 cm^−1^~400 cm^−1^ with 64 scans to investigate the modification mechanism and anti-aging effect.

#### 4.4.4. Gel Permeation Chromatography Test

Gel Permeation Chromatography was used to compare the relative molecular weight and the distribution state of relative molecular weight before and after aging as a measure of the high temperature stability and aging resistance of asphalt [35,36]. In this study, Waters 1515 gel permeation chromatography was used, the mobile phase was tetrahydrofuran, the flow rate was 1.0 mL/min, the concentration of the sample solution was 2.0 mg/mL, the injection volume was 50 uL, and the testing time was 30 min.

## Figures and Tables

**Table 1:** Results of various performance tests.

Number	A: RP Dosage (%)	B: SOM-LDHs Content (%)	C: Shearing Temperature (°C)	Needle Penetration (0.1 mm)	Ductility at 10 °C (cm)	Softening Point (°C)
1	18	2	175	54.3	17.9	51.7
2	22	2	175	49.5	20.1	55.5
3	22	4	160	47.9	19.9	56.9
4	20	6	160	50.7	22.2	56.1
5	18	6	175	52.3	15.4	54.3
6	20	2	190	52.9	24.9	54.4
7	20	4	175	52.9	23.5	55.5
8	20	4	175	52.4	23.4	55.2
9	18	4	190	53.7	16.9	52.9
10	18	4	160	53.1	16.2	52.1
11	20	4	175	53	23.3	55.5
12	22	6	175	46.4	19.4	59.4
13	20	4	175	52.7	23.2	55.2
14	20	6	190	51.1	22.9	56.9
15	22	4	190	47.2	20.2	57.2
16	20	4	175	52.9	23.1	55.3
17	20	2	160	52.1	24.6	53.1

**Table 3:** Short-term aging performance index of three kinds of asphalt.

Experimental Parameters		Asphalt	RP Modified Asphalt	RP/SOM-LDHs Composite Modified Asphalt
Before aging	25°C needle penetration (0.1 mm)	65.2 ± 1.2 (63.2–67.2)	50.2 ± 0.8 (48.8–51.6)	47.8 ± 0.6 (46.8–48.8)
	Viscosity (135°C, Pa·s)	0.457 ± 0.012 (0.436–0.478)	2.43 ± 0.07 (2.30–2.56)	2.88 ± 0.09 (2.73–3.03)
	Quality (g)	34.962 ± 0.015 (34.932–34.992)	35.162 ± 0.012 (35.140–35.184)	35.171 ± 0.010 (35.154–35.188)
After aging	Quality (g)	26.783 ± 0.022 (26.745–26.821)	32.145 ± 0.018 (32.113–32.177)	33.156 ± 0.015 (33.129–33.183)
	Mass variation (%)	0.234 ± 0.010 (0.217–0.251)	0.086 ± 0.008 (0.072–0.100)	0.057 ± 0.006 (0.047–0.067)
	Residual needle penetration ratio (%)	68.4 ± 1.5 (65.7–71.1)	75.1 ± 1.2 (72.7–77.5)	84.1 ± 1.0 (82.2–86.0)
	Viscosity ratio	1.66 ± 0.04 (1.58–1.74)	1.23 ± 0.03 (1.18–1.28)	1.08 ± 0.02 (1.04–1.12)
	Viscosity (135 °C, Pa·s)	0.759 ± 0.018 (0.727–0.791)	3.06 ± 0.08 (2.92–3.20)	3.12 ± 0.08 (2.98–3.26)
	25 °C needle penetration (0.1 mm)	44.6 ± 1.0 (42.8–46.4)	37.7 ± 0.9 (36.0–39.4)	40.2 ± 0.8 (38.6–41.8)

**Table 4:** Roughness values of different types of asphalt.

Asphalt Type	Roughness (nm)		ISAD (%)
	R_q_	R_a_	
RP/SOM-LDHs composite modified asphalt	1.81	1.12	0.053
Asphalt	5.73	2.83	0.218
RP-modified asphalt	1.99	1.26	0.054

**Table 5:** Calculation results for each asphalt.

Asphalt Sample	Index of *I_C=O_*			Index of *I_S=O_*		
	Unaged	UV Aging	Rate of Change	Unaged	UV Aging	Rate of Change
Asphalt	0.00572	0.04846	7.47203	0.05613	0.09221	0.64279
RP-modified asphalt	0.00628	0.04204	5.69427	0.05689	0.08629	0.51679
RP/SOM-LDHs composite modified asphalt	0.00691	0.03256	3.71201	0.05879	0.08543	0.45314

**Table 6:** *M_n_*, *M_w,_* and PDI calculation results of different types of asphalt.

Asphalt Sample	Before UV Aging			After UV Aging			The Change Rate of *M_w_* Before and After UV Aging (%)
	*M_w_*	*M_n_*	PDI	*M_w_*	*M_n_*	PDI	
Asphalt	2026	590	3.43	2497	564	4.43	23.25
RP-modified asphalt	2071	592	3.50	2492	552	4.51	20.33
RP/SOM-LDHs composite modified asphalt	2097	589	3.56	2501	551	4.54	19.27

**Table 7:** Technical index of 70# asphalt.

Performance Index		Unit	Test Results	Technical Requirements	Test Methods
Needle penetration (25 °C, 100 g, 5 s)		0.1 mm	65.2	60~80	T0640
Dctility (5 cm/min, 10 °C)		cm	30.4	≥20	T0605
Softening point (global method)		°C	51.0	≥46	T0606
Flash point		°C	270	≥260	T0611
Density (25 °C)		g/cm^3^	1.159	Measured value	T0603
After RTFOT	Mass variation	%	0.234	−0.8~+0.8	T0610
	Residual penetration ratio	%	68.4	≥61	T0604
	Dctility (5 cm/min, 10 °C)	cm	16.5	≥20	T0605

**Table 9:** Performance index of rubber powder.

Test Items	Relative Density	Tenor (%)	Rubber Hydrocarbon Content (%)	Ash (%)	Sieve Residue (%)
Test result	1.2	0.03	58	3.3	2.6

**Table 12:** Outdoor indoor ultraviolet radiation time conversion.

Outdoor Aging Time (Months)	Indoor Simulation Time (Hours)
6	172.8
12	345.6

## Data Availability

All data that support the findings of this study are included within the article.
